# Targeting the Blood–Brain Tumor Barrier with Tumor Necrosis Factor-α

**DOI:** 10.3390/pharmaceutics14071414

**Published:** 2022-07-06

**Authors:** Angelo Corti, Teresa Calimeri, Flavio Curnis, Andres J. M. Ferreri

**Affiliations:** 1 Tumor Biology and Vascular Targeting Unit, Division of Experimental Oncology, IRCCS San Raffaele Scientific Institute, 20132 Milan, Italy; curnis.flavio@hsr.it; 2 Faculty of Medicine, Università Vita-Salute San Raffaele, 20132 Milan, Italy; 3 Lymphoma Unit, Department of Onco-Hematology, IRCCS San Raffaele Scientific Institute, 20132 Milan, Italy; calimeri.teresa@hsr.it

**Keywords:** blood–brain tumor barrier, permeabilization, brain tumors, primary central nervous system lymphoma, tumor necrosis factor-alpha, targeted delivery, vascular targeting, CD13, TNF, TNF receptors, NGR-TNF

## Abstract

The blood–brain tumor barrier represents a major obstacle for anticancer drug delivery to brain tumors. Thus, novel strategies aimed at targeting and breaching this structure are of great experimental and clinical interest. This review is primarily focused on the development and use of a derivative of tumor necrosis factor-α (TNF) that can target and alter the blood–brain-tumor-barrier. This drug, called NGR-TNF, consists of a TNF molecule fused to the Cys-Asn-Gly-Arg-Cys-Gly (CNGRCG) peptide (called NGR), a ligand of aminopeptidase N (CD13)-positive tumor blood vessels. Results of preclinical studies suggest that this peptide-cytokine fusion product represents a valuable strategy for delivering TNF to tumor vessels in an amount sufficient to break the biological barriers that restrict drug penetration in cancer lesions. Moreover, clinical studies performed in patients with primary central nervous system lymphoma, have shown that an extremely low dose of NGR-TNF (0.8 µg/m^2^) is sufficient to promote selective blood–brain-tumor-barrier alteration, increase the efficacy of R-CHOP (a chemo-immunotherapy regimen) and improve patient survival. Besides reviewing these findings, we discuss the potential problems related to the instability and molecular heterogeneity of NGR-TNF and review the various approaches so far developed to obtain more robust and homogeneous TNF derivatives, as well as the pharmacological properties of other peptide/antibody-TNF fusion products, muteins and nanoparticles that are potentially useful for targeting the blood–brain tumor barrier. Compared to other TNF-related drugs, the administration of extremely low-doses of NGR-TNF or its derivatives appear as promising non-immunogenic approaches to overcome TNF counter-regulatory mechanism and systemic toxicity, thereby enabling safe breaking of the BBTB.

## 1. Introduction

The prognosis of patients with tumors of the central nervous system (CNS), such as primary lymphomas, gliomas, or brain metastases from neoplasms that originate in other organs, is still poor. This is mainly due to the presence of the blood–brain barrier (BBB), which represents an important obstacle for anticancer drug delivery to brain tumors. Although tumors in the brain compromise the integrity of the BBB in the affected area, this barrier (referred to as the “blood–brain tumor barrier”, BBTB) is heterogeneously permeable and represents, therefore, a major obstacle for a homogeneous penetration of anticancer drugs in tumor tissues. The description of structure and function of the BBTB is beyond the scope of this paper, and we refer the readers to an excellent review on this subject, recently published [1]. Thus, patients with tumors affecting the CNS, primary or secondary, would greatly benefit from the development of new tools capable of altering the BBTB and, consequently, enhancing the penetration of anticancer drugs in tumor tissues. The various approaches so far developed to meet this medical need have been recently reviewed [2]. These strategies include approaches based on (a) direct delivery of drugs into the brain parenchyma (e.g., by intraoperative placement of the drug or by convection-enhanced delivery), (b) exploitation of normal transport mechanisms (e.g., by solute carrier-mediated or receptor-mediated transcytosis), and (c) use of tools that increase the BBTB permeability (e.g., osmotic agents, focused ultrasounds, or pharmacological means) [2]. Another strategy recently developed for delivering nanoparticles to brain tumors exploits, in a counter-intuitive manner, the impermeability of the BBB in a two-step approach based on the selective retainment of ligand molecules on the surface of the brain vasculature, thanks to the lower endocytic rate of brain endothelium compared to peripheral endothelial cells, followed, in a second step, by administration of ligand-selective nanoparticles [3]. However, despite the various strategies so far developed, overcoming the BBTB still represents a major problem for brain tumor therapy. The present review is focused on the development and use of NGR-TNF, a genetically engineered derivative of tumor necrosis factor-α (TNF) that can target the tumor blood vessels and increase the permeability of the BBTB. We review here the structural and functional properties of this drug (which consists of TNF fused to the C-terminal residue of the CNGRCG peptide), its receptors, its biological effects on the barriers that limit drug penetration in solid tumors, and its mechanisms of action. In addition, we review the results of preclinical and clinical studies performed with this drug, alone or in combination with chemo- and immuno-therapy, including those of a recent study performed in patients with primary CNS lymphomas (PCNSL) showing that this drug may represent a valuable tool for breaching the BBTB. Finally, we also discuss the problems related to NGR-TNF instability and review the approaches so far developed to obtain more robust TNF derivatives, as well as the potential advantages/disadvantages of other peptide/antibody-TNF conjugates, muteins and TNF-based nanodrugs that could be potentially used for targeting brain neoplasms and other solid tumors.

## 2. The CNGRCG-TNF Recombinant Protein (NGR-TNF) and Its Receptors

TNF is an inflammatory cytokine originally discovered for its capability to cause massive hemorrhagic necrosis of cancer lesions in mice [4,5]. Unfortunately, despite the impressive anti-cancer effects observed in mice, studies performed in cancer patients in the late 1980s showed that TNF, upon systemic administration, can induce toxic effects and no, or very modest, anti-tumor responses [6,7]. However, clinical studies performed in the 1990s showed that locoregional administration of a high doses of TNF combined with chemotherapy, by isolated limb perfusion, can induce high response rates in subjects with sarcomas of the extremities or with melanomas [8,9,10,11]. These results indicate that TNF can be used in a safe and successful manner to treat cancer patients if high levels of cytokine are attained in tumors, locally and not in circulation. These notions stimulated, in the subsequent years, studies aimed at developing targeting strategies for this cytokine, in the attempt to enable systemic administration of therapeutically efficacious doses of TNF without causing toxicity. Among the various approaches that have been pursued for this purpose, targeted delivery of TNF to the tumor-associated vessels (e.g., by fusing this cytokine with peptide or antibody ligands that recognize receptors or molecules expressed by the tumor vasculature) represents a valid strategy [12]. NGR-TNF, a recombinant homo-trimeric protein consisting of CNGRCG fused to the N-terminal residue of TNF and produced in *E. coli*, represents a prototypical example of these class of compounds [13].

The CNGRCG peptide was originally identified by panning peptide-phage libraries in tumor-bearing mice [14]. This compound contains two cysteines and a disulfide-constrained NGR motif that can recognize a specific form of CD13 (a membrane metalloprotease called aminopeptidase N) barely (or not at all) expressed by blood vessels in normal conditions and up-regulated in the angiogenic vasculature [15,16,17,18]. In solid tumors this protease is expressed by pericytes and endothelial cells, and, in certain cases, also by fibroblasts and cancer cells. CD13 is also expressed by cells of healthy tissues, such as epithelial cells of the prostate, small intestine, kidney, liver, as well as by mast cells, myeloid cells, antigen-presenting cells, and keratinocytes [19,20,21,22]. CD13 is highly expressed by pericytes in the brain vasculature, i.e., on cells that are behind the endothelial barrier and, therefore, poorly accessible to circulating molecules in normal tissues, but potentially accessible in those areas of brain tumors with an altered BBTB. Notably, a recent study performed in PCNSL patients have revealed CD13 expression on the tumor endothelium, i.e., on cells that are accessible to circulating molecules also in areas with an intact BBTB [23,24]. Experimental evidence suggests that compounds containing the CNGRCG sequence can recognize CD13^+^ tumor vasculature, but not other normal tissues that express high levels of CD13 [13,16].

The molecular bases of this selective recognition are still unclear. CD13 is a dimeric glycoprotein with an archlike structure and with monomers that can assume closed or open conformations [25]. It is possible that different conformations in different tissues, owing to the presence of tissue-specific cofactors, post-translational modifications or signaling molecules, may result in a differential binding affinity for NGR-containing peptides. According to this view, studies on the interaction of a cyclic CNGRC peptide with various CD13-expressing cells (endothelial cells, pericytes, tumor cells, and myeloid cells), by 2D Transferred Nuclear Overhouser Effect Spectroscopy (2D TR-NOESY) and by immunofluorescence techniques, showed that the expression of CD13 was necessary but not sufficient for the binding, as binding was observed to cells that form the tumor vasculature (endothelial cells and pericytes) and to some tumor cell lines, but not to myeloid cells [26].

Because of their tumor vasculature homing properties, CNGRCG and other NGR-peptides have been exploited for delivering a variety of therapeutic or imaging compounds to neoplastic lesions, including liposomes, chemotherapeutic agents, DNA, viruses, anti-angiogenic compounds, fluorescent molecules, contrast agents and also cytokines [12]. In NGR-TNF, which was the first NGR-cytokine conjugate to be developed, the CNGRCG domain does not impair folding, TNF subunit oligomerization and binding to type-1 and type-2 TNF receptors. Furthermore, the TNF domain does not impair the interaction of NGR with its receptor (CD13) [13]. Thus, NGR-TNF can engage high-avidity interactions with TNF-receptors and CD13 on cells that express both types of receptors, as it occurs in endothelial cells of the tumor vasculature, but not in normal vessels (which lack CD13) [13]. For the same reason NGR-TNF is unlikely to engage high-avidity multivalent interactions with the soluble CD13, alone, present in the blood. The high-avidity interactions with both types of receptors in the tumor vasculature can likely account for the tumor-homing properties of NGR-TNF and its capability to exert anti-cancer effects at extremely low doses in mice [27]. This view is supported by the observation that the anti-cancer activity of low-dose NGR-TNF in murine models is abrogated by co-administration of a neutralizing anti-CD13 antibody or by anti-TNF-Rs antibodies [13,27]. However, it is also worth highlighting the fact that the function of CD13 is not limited to NGR-TNF homing to tumor vessels, as upon NGR-TNF binding this membrane protein can also induce co-signaling mechanisms that impair the activation of pro-survival pathways induced by the TNF moiety (Ras, Erk, Akt, NF-kB) without affecting other pathways related to stress and cell death (p38 and JNK), a mechanism that may contribute to the overall biological activity of the fusion protein [26]. In other words, both NGR and TNF domains have a direct role in both cell binding and signaling. Whether or not TNF receptors are internalized, recycled or destroyed after NGR-TNF binding and whether the NGR domain of TNF is cleaved upon binding to CD13 are unknown.

Finally, considering the notion that CD13 is expressed in angiogenic vessels, it is important to highlight the fact that besides targeting the angiogenic vasculature of tumors, off-target delivery of NGR-TNF to non-tumoral angiogenic vessels might occur in the case of concomitant diseases with an angiogenic component.

## 3. Preclinical Studies of NGR-TNF

### 3.1. NGR-TNF as a Single Agent in Murine Models of Solid Tumors

Considering that human TNF can efficiently bind murine type-1, but not type-2, TNF receptor [28], most preclinical studies in mice have been performed with murine NGR-TNF. Studies in murine models of lymphoma, fibrosarcoma, and melanoma have shown that the dose–response curve of systemically administered NGR-TNF is markedly different from that of TNF: while both TNF and NGR-TNF have therapeutic anti-cancer activity at doses in the microgram range, only NGR-TNF can induce anti-tumor responses when administered at doses in the picogram range (e.g., 100 pg/mouse). Nanogram doses of NGR-TNF (e.g., 3–10 ng) were paradoxically less active than picograms, pointing to a triphasic-dose–response curve [13,27]. Mechanistic studies have shown that this behavior depends on the shedding of soluble TNF-receptors in circulation (i.e., of TNF inhibitors), which can be triggered by TNF and NGR-TNF at doses >1 ng/mouse. This counter-regulatory mechanism efficiently inhibits the potential anti-cancer and toxic effects of both TNF and NGR-TNF [27]. When doses in the range of micrograms are used, however, both drugs can overcome these counter-regulatory mechanisms and induce strong anti-cancer effects, but, for the same reason, they can also cause systemic toxic reactions. In contrast, doses of NGR-TNF in the range of picograms do not induce the shedding of soluble TNF receptors and can exert pharmacological effects without causing systemic toxicity [27]. Thus, the use of extremely low doses of NGR-TNF may represent a strategy to avoid toxicity as well as systemic counter-regulatory mechanisms.

Studies on the mechanisms of NGR-TNF anti-tumor activity have shown that targeted delivery of low amounts of this drug to tumor blood vessels (e.g., 100 pg/mouse, >100,000-fold lower than the median lethal dose, LD50) can affect the tumor vasculature and microenvironment, causing the up-regulation of intercellular adhesion molecule (ICAM)-2 and vascular cell adhesion molecule (VCAM)-1 in the endothelial lining of tumor vasculature and the release of various chemokines and cytokines involved in the activation and migration T-cells in the tumor tissue, such as MCP-1/CCL-2, MIP-2, oncostatin-M, MCP-3/CCL-7, and stem cell factor [29]. Low-dose NGR-TNF, but not TNF, can affect VE-cadherin dependent adherence junctions and enhance vascular permeability in tumors [30]. Accordingly, magnetic resonance imaging in lymphoma-bearing mice, 2 hours after drug administration, have shown an enhanced leakage of the blood pool contrast agent from tumor blood vessels [12]. Low-dose NGR-TNF can also cause apoptosis of endothelial cells and, at later time points, also apoptosis of tumor cells, likely because of vascular damage and nutrients/oxygen deprivation [12]. It appears, therefore, that a combination of endothelial barrier alteration, vascular damage and inflammatory/immune responses contribute to the overall anti-cancer effects of low-dose NGR-TNF.

### 3.2. Effect of Low-Dose NGR-TNF on the Barriers That Limit the Penetration of Chemotherapeutic Drugs in Tumors

Low-dose NGR-TNF (100 pg/mouse, systemically administered) can increase the response of tumors to chemotherapeutic drugs, as observed with cisplatin, melphalan, doxorubicin, gemcitabine and paclitaxel in various models of transplantable tumors and in an orthotopic model of prostate cancer (TRAMP) [27,31,32].

**Figure 1:**
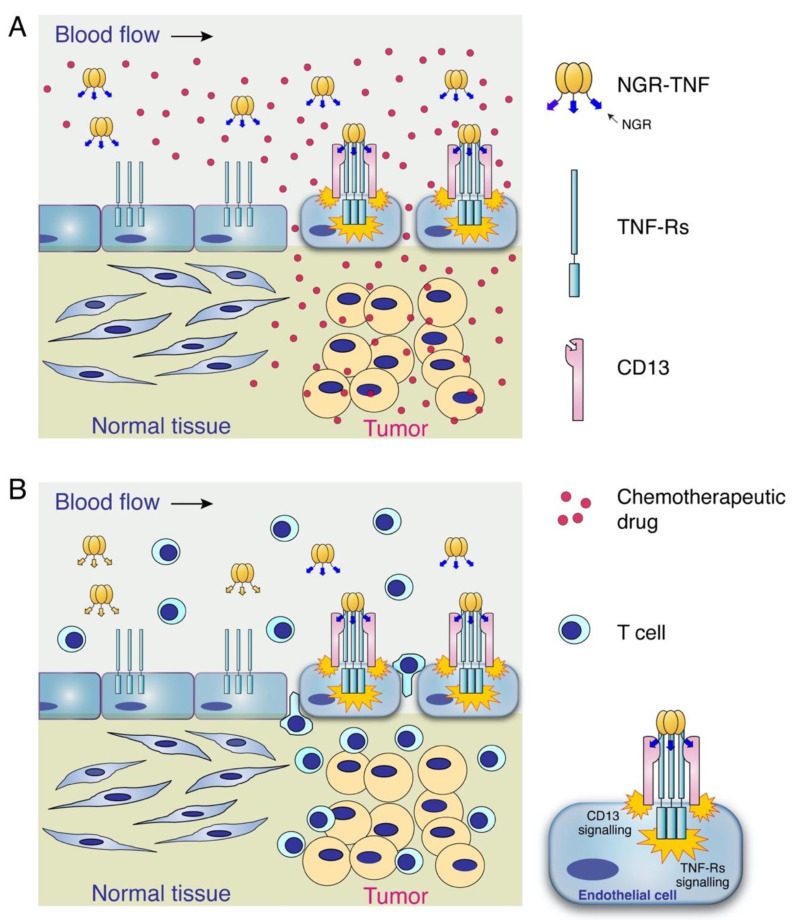
Schematic representation of the primary mechanisms of the synergistic effects of low-dose NGR-TNF with chemo/immunotherapy. Low-dose NGR-TNF recognizes with high avidity CD13 and TNF receptors (TNF-Rs) expressed in the vasculature of solid tumors. This interaction activates TNF-Rs- and CD13-dependent signaling mechanisms that lead to selective activation of endothelial cells and, consequently, to alteration of the endothelial-barrier function and cytokine-chemokine secretion. These mechanisms favor chemotherapeutic drug penetration (**A**) and CD8 T-cells infiltration (**B**) in tumor tissues. These effects are also followed, at later time points, by vascular damage (see text). The selectivity of these effects depends on the fact that CD13 (the NGR receptor) is overexpressed in tumor vessels and little, or not at all, by endothelial cells in normal tissues.

Maximal synergism can be obtained with a 2-hour delay between NGR-TNF and chemotherapeutic drug administrations, irrespective of tumor model and drug used. Mechanistic studies performed in mice bearing melanomas or lymphomas have shown that low-dose NGR-TNF can increase the tumor uptake of the anthracycline doxorubicin [27]. This phenomenon can be related to the capability of TNF to reduce the barriers that limit drug penetration in tumor tissues, such as those related to the endothelial barrier function and to the high interstitial pressure, thereby leading to an increase of the convective transport of drugs through the tumor vasculature wall, at least in those areas that are poorly perfused and characterized by low permeability [9,33,34,35,36,37,38,39,40]. Notably, chromogranin A, a neurosecretory glycoprotein capable of inhibiting the vascular leakage induced by TNF, can also inhibit the synergism between NGR-TNF and chemotherapeutic drugs in tumor-bearing mice [30]. This observation is in line with the hypothesis that an increased vascular permeability is a crucial mechanism of the increased chemotherapeutic-drug penetration in tumors, after NGR-TNF administration, and synergism (Figure 1A). NGR-TNF and doxorubicin synergism occurs in immunocompetent mice, but not in interferon-γ (IFNγ)– knock-out or nude mice, suggesting that a fully functional immune system and IFNγ are necessary for the overall anti-tumor effects of this combination [41].

### 3.3. Effect of Low-Dose NGR-TNF on the Infiltration of Lymphocyte in Tumors

Low-dose NGR-TNF can promote lymphocyte extravasation in tumors [29] (Figure 1B). This phenomenon is likely related to the capability of NGR-TNF to loosen vascular endothelial cadherin-dependent adherence junctions, to up-regulate leukocyte-adhesion molecules on the tumor vasculature, and to promote the secretion of various chemokines in neoplastic tissues (see above paragraphs). Studies performed in murine models of melanoma and in a spontaneous-orthotopic model of prostate cancer (TRAMP) have shown that NGR-TNF can increase tumor-tissue infiltration of either endogenous or adoptively transferred cytotoxic T-cells, without modification of T-cell distribution in blood, spleen or kidney in tumor-bearing mice [29]. Notably, NGR-TNF can increase the efficacy of adoptive or active immunotherapy of tumor-bearing mice, either when administered alone or in combination with chemotherapy [29]. Comparable doses of TNF were marginally or not at all active in the same models, suggesting that the NGR-directed targeted delivery of TNF to the tumor vasculature was necessary for the efficacy of the combined therapies. Low-dose NGR-TNF can also increase the efficacy of TCR-engineered tumor-redirected lymphocytes, leading to tumor eradication in the orthotopic model of prostate cancer [42]. Finally, studies performed in mice bearing prostate cancers or melanomas, have shown that low doses of NGR-TNF can enhance the infiltration of effector T cells in tumors, as well as the therapeutic effects of immune checkpoint blockers combined with adoptive cell therapy [43].

## 4. Clinical Studies with NGR-TNF

### 4.1. Clinical Studies with NGR-TNF in Patients with Solid Tumors

Human NGR-TNF (NGR-hTNF) has been evaluated alone or in combination with chemotherapy in several phase-I and -II studies enrolling patients with various types of solid tumors, and in also in a phase-III study in patients with malignant pleural mesothelioma.

The first phase I study, conducted on 69 patients with histologic or cytologic confirmation of advanced cancer, showed for the first time in humans that treatment with NGR-hTNF could cause anti-vascular effects in tumors [44]. NGR-hTNF was administered once every 3 weeks by a 20 to 60 min intravenous infusion using escalating doses (0.2–60 µg/m^2^) with a maximum tolerated dose (MTD) of 45 μg/m^2^ administered in 1 hour. The most frequent observed toxicities were rigors and fever. The terminal half-life of NGR-hTNF was 1 to 2 hours. No objective responses were observed, but 39% of patients had stable disease (SD), with a median duration of response of 12 weeks. Based on the results of another phase-I study, designed to define safety and optimal dose in patients with advanced solid tumors, the dose of 0.8 µg/m^2^ was chosen for subsequent studies, either as single-agent or in combination with chemotherapeutic drugs, considering the favorable toxicity profile observed with this dose, the disease control obtained (44% of SD for a median time of 5.9 months), the lack of stimulation of TNF-receptors shedding and the significant anti-vascular effects observed [45]. Another important Phase I clinical study, performed on 31 patients with advanced solid tumors, showed that the anti-vascular activity of NGR-hTNF was inversely correlated to the tumor dimension, probably because of the presence of a less-mature neovasculature in small lesions [46]. This study also confirmed that the levels of circulating soluble TNF-receptors were not increased by low doses of NGR-hTNF (<1.3 µg/m^2^) and showed that patients did not develop anti-NGR-hTNF antibodies.

Thus, phase II clinical studies with low-dose NGR-TNF (0.8 µg/m^2^, 1 h infusion, weekly or every 3 weeks) as a single agent were then conducted in pre-treated patients with hepatocellular carcinoma (HCC), malignant pleural mesothelioma (MPM), and colorectal cancer (CRC). The results showed again anti-vascular effects and significant disease control [47,48,49]. The treatment was well-tolerated, as no drug-related grade 3 to 4 toxicities were registered except for 1 grade 3 syncope in the 3-weekly cohort of the MPM study.

Phase I studies on the combination of low-dose NGR-hTNF with doxorubicin (60–75 mg/m^2^) [50] or cisplatin (80 mg/m^2^) [51] showed that these therapeutic regimens were feasible and well-tolerated. Notably, a promising anti-tumor activity was registered in patients pretreated and refractory to the chemotherapeutic drug used in the combination. Low-dose NGR-hTNF, combined with doxorubicin, was also evaluated in a phase II study enrolling 37 relapsed ovarian cancer patients with a platinum-free interval lower than 12 months. The overall response rate was 66% (23% PR and 43% SD), median PFS and OS were 5 months and 17 months, respectively [52]. No unexpected toxicities and strong association between baseline-lymphocyte counts and outcomes were reported. Notably, this association was observed also in unselected patients with relapsed small cell lung cancer injected with NGR-hTNF and doxorubicin [53]. In these patients, similar anti-tumor activity was observed in both platinum-sensitive and platinum-resistant patient cohorts. In another phase-II study, two sequential groups of CRC patients who had failed standard treatment received NGR-hTNF, 0.8 or 45 μg/m^2^, combined with a fixed dose of capecitabine-oxaliplatin (XELOX). Both these treatments were safe, but apparently a sign of efficacy was evident only with the lower NGR-hTNF dose [54]. This is a remarkable observation, as it supports the concept that “the-more-is-better” principle is not valid for NGR-hTNF (and likely also for other TNF derivatives). As observed in preclinical studies, the lower anti-tumor activity of high-dose NGR-hTNF (45 μg/m^2^) seemed to be related to the induction of soluble TNF-receptors shedding in the circulation, i.e., the release of TNF inhibitors. However, the results of a phase I study conducted with doses (60–325 μg/m^2^) higher than the MTD previously established in 48 patients with refractory solid tumors (45 μg/m^2^) suggest that these doses are tolerated and can induce anti-tumor effects if a more protracted length of infusion (2 h) and mild premedication with paracetamol are used [55]. This behavior observed in patients recapitulates the complex dose–response curve observed in murine models (discussed above).

NGR-hTNF was also combined with a peptide-based vaccination in a pilot phase-I study enrolling patients with metastatic melanoma [56]. Accrual was slow and the trial was closed earlier due to new therapies that became available for metastatic melanoma. However, the combination was associated with an ex-vivo T-cell response and a long-term overall survival (>4 months in six out of eight evaluable patients).

The results of a randomized, double blind, placebo-controlled phase-III clinical trial on 400 previously treated patients with MPM and treated with or without low-dose NGR-hTNF (0.8 μg/m^2^, weekly) in combination with the best investigator choice have been published in 2018 [57]. Although the primary end point on the overall survival (OS) was not reached for the entire population of patients treated with NGR-hTNF (*n* = 200) a significant improvement in progression-free survival (PFS) and OS was observed in patients who progressed more rapidly after first-line treatment (short treatment-free-interval, representing 50% of the entire patient population), suggesting that there could be a benefit from treating patients with more aggressive MPM with NGR-hTNF.

### 4.2. Targeting the Blood–Brain Tumor Barrier with NGR-TNF in Patients with PCNSL

**Figure 2:**
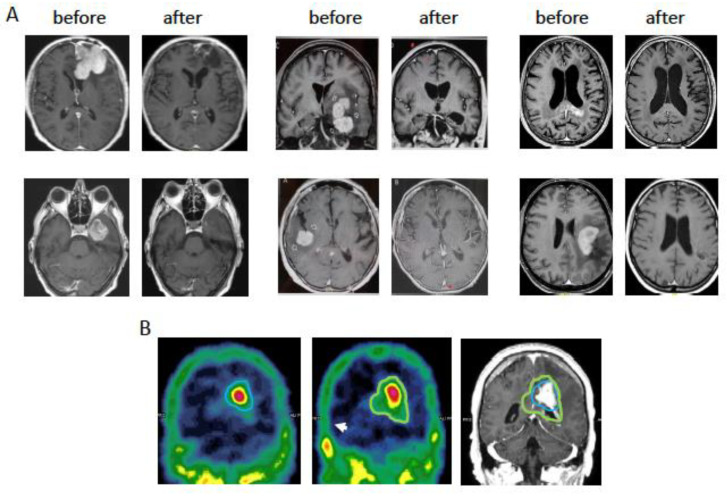
Examples of responses to NGR-hTNF/R-CHOP combination in patients with PCNSL. (**A**) DCE-MRI (gadolinium-enhanced T1-weighted scan) before and after treatment with 2–4 cycles of R-CHOP preceded by NGR-hTNF in 6 PCNSL patients. (**B**) SPECT analysis of a patient before and after the third treatment with NGR-hTNF and R-CHOP, showing an increase of uptake of 99mTc-DTPA. The volume of ≥30% uptake of 99mTc-DTPA is contoured in the two studies performed before (left image, blue line) and after (central image, green line) administration of NGR-hTNF/R-CHOP. The contoured volumes are also reported on the image obtained by gadolinium-enhanced T1-weighted MRI (right image) showing the tumor location. The volume of increased uptake before and after NGR-hTNF/R-CHOP administration was 22 and 40 cm^3^, respectively. Part of this research was originally published in *Blood* [24] and *Blood Advances* [23].

Primary diffuse large B-cell lymphoma (DLBCL) of the CNS (PCNSL), a rare extra-nodal non-Hodgkin lymphoma confined to brain, eyes, meninges, and other structures of the CNS, is a paradigmatic example of one of the major challenge in the treatment of CNS tumors: the delivery of drugs across the BBB [58]. PCNSL are usually treated with high-dose methotrexate-based combinations, which require hospitalization and large expertise [59]. The use of R-CHOP (rituximab, cyclophosphamide, doxorubicin, vincristine, and prednisone, the standard treatment of systemic DLBCL) might overcome these difficulties, but CNS penetration of these drugs is poor [58]. Thus, strategies aimed at improving drug penetration in this setting are of great interest and many invasive and noninvasive approaches have already been tested [58]. Studies performed in mice bearing brain metastases of breast cancer have shown that systemic administration of relatively high doses of TNF can induce BBTB permeabilization [23] and enhance tumor penetration of therapeutic compounds [60], but, as discussed above, this approach cannot be used in patients because of its inherent systemic toxicity [12]. Based on the idea that these limitations could be overcome by BBTB permeabilization with low-dose NGR-hTNF followed by R-CHOP, a phase-II trial on NGR-hTNF plus R-CHOP in patients with relapsed/refractory PCNSL has been designed and conducted (“INGRID” trial). The trial was designed to include two distinct parts: (a) an exploratory phase on the first 10 enrolled patients, aimed at assessing the feasibility of the NGR-hTNF/R-CHOP combination and obtaining the “proof of principle” of BBTB alteration by NGR-hTNF [24]; and (b) an expansion phase on 28 patients, aimed at assessing the efficacy and safety of this therapy in the whole patients cohort [23]. Treatment schedule consisted of six courses of R-CHOP21 preceded by NGR-hTNF (0.8 µg/m^2^ delivered 2 hours before R-CHOP by a 1 h infusion). Per protocol, the first course of R-CHOP was not preceded by NGR-hTNF in the first 10 patients. All subjects who achieved a partial response (PR) or a complete response (CR) at the end of the program, were evaluated for consolidative therapy which, per protocol and accordingly to prior treatments, could be represented by whole-brain radiation therapy (30–36 Gy), carmustine-thiotepa-conditioned autologous stem cell transplantation or oral lenalidomide maintenance. A total of 28 patients were enrolled (median age: 58 years, range 26–78; 14 males); most patients had unfavorable features at trial registration (IELSG risk score intermediate or high in 82%). Tumor response was observed in 21 patients (75%; 95%CI = 59–91), complete in 11 patients, and the predetermined efficacy threshold (≥12 responses) was achieved, indicating that the combination of NGR-hTNF and RCHOP was efficacious in these patients (Figure 2A). At a median follow-up of 34 months (27–44 months), six patients were still alive, and five patients remained relapse free. Of note, the responses observed after R-CHOP alone in the first 10 enrolled patients were not significant, with most patients showing stable disease or progressive disease, excluding, *bona fide*, that responses achieved later were exclusively due to immune-chemotherapy activity. Treatment was well-tolerated. Toxicities were quickly solved with no need of dose reductions or interruptions, and, overall, no treatment related death was observed. A total of 15 systemic adverse events were recorded in 11 patients, with 9 grade 1–2 reactions to NGR-hTNF infusion. Dynamic-contrast enhanced magnetic resonance imaging and single-photon emission computed tomography studies showed an increase of vascular permeability after NGR-hTNF infusion in tumor and perilesional areas (Figure 2B). Notably, immunohistochemical analysis of tumor tissue sections have revealed the presence of CD13 on the luminal side of tumor vasculature in all diagnostic brain biopsies evaluated, suggesting that the NGR receptor target was accessible to NGR-hTNF intravenously delivered [23,24]. The localized action of TNF on tumoral and peritumoral areas, likely mediated by CD13 targeting, is also suggested by the observation that no changes in the cerebrospinal fluid/plasma drug levels occurred after NGR-hTNF infusion. The effect of NGR-hTNF on the blood–retina barrier and the blood–cerebrospinal fluid barrier remains to be established because none of the patients had meningeal disease at trial registration, and only three patients had ocular involvement. Overall, the results obtained in PCNSL patients are in line with the good tolerability of NGR-hTNF observed in previous studies on patients with other solid tumors, thus suggesting that this innovative therapeutic approach deserves to be investigated as first-line treatment in PCNSL patients.

### 4.3. NGR-TNF Immunogenicity

Studies on the immunogenicity of the CNGRCG domain of NGR-TNF, carried out in rabbits and mice injected with NGR-TNF or with various compounds consisting of CNGRCG coupled with immunogenic carrier proteins, have shown that the CNGRCG peptide ligand is poorly, or not at all, immunogenic [61]. Experiments based on molecular dynamics simulation predicted that the most populated structures of CNGRC are superimposable to a GNGRG hairpin structure present in human fibronectin 5th type I repeat (FN-I5) [61]. This can explain the low immunogenicity of the CNGRCG peptide sequence, as it likely mimics a “self” structure. Accordingly, as discussed above, no anti-NGR-TNF antibodies were raised in patients treated with human NGR-TNF, even after repeated injections.

### 4.4. NGR-TNF Derivatives with Improved Stability and Homogeneity

TNF is a homotrimeric protein. Structural studies of human and murine NGR-TNF, produced by genetic engineering technology in *Escherichia coli* (*E. coli*) cells, have shown that these products consist of trimers made by subunits with different molecular weights, including subunits with the expected molecular weight (17939.39 Da and 17844.25 Da, respectively) and subunits with −17, +1, +42 and +58 Da than expected [62]. The molecular heterogeneity of human and murine NGR-TNF is related to post-translational modifications of their N-terminal domains, including N-terminal acetylation and oxidation (+42 and +58 Da), asparagine deamidation (+1 Da), and formation of 6–7 membered rings between cysteine α-amino group and asparagine side-chain (−17 kDa). These post-translational modification and degradation reactions need a cysteine (C) in the first position (with a free α-amino group) and an asparagine (N) in second position, as in the CNGRCG domain of NGR-TNF [62].

While the acetylated-CNGRCG domain can recognize CD13 with a binding strength similar to that of the non-acetylated domain, the −17 Da subunits are likely non-functional, as this modification (which involves the reaction of the cysteine α-amino group with the asparagine side-chain of the CNGRCG domain) destroys the NGR motif [62]. Thus, incorporation of these altered subunits in NGR-TNF may have important pharmacological implications.

The asparagine deamidation reaction leads to the formation of aspartate and isoaspartate. The consequent NGR-to-isoDGR transition causes receptor switch from CD13 to αvβ3 integrin, isoDGR being an integrin-binding motif [63]. Notably, αvβ3 is an integrin overexpressed in the tumor neovasculature and for this reason isoDGR-TNF (a degradation product of NGR-TNF) is endowed of potent anti-cancer activity [63]. It is possible, therefore, that the asparagine deamidation reaction (which may occur in low but significant amounts in vitro, during NGR-TNF production and storage, and in vivo, after drug injection in patients) may lead to a dual mechanism of tumor vessel targeting based on CD13 and αvβ3 recognition. The effect of isoDGR-TNF on other αvβ3-expressing cells, such as platelets and myeloid cells (dendritic cells, macrophages, microglia) is unknown.

Considering the molecular heterogeneity of NGR-TNF subunits and assuming a random association of different subunits in trimer formation, different trimers are expected to be present in NGR-TNF preparations. This may represent a major problem in drug development, particularly in terms of lot-to-lot consistency and quality assurance. Thus, various attempts have been made to generate NGR-TNF derivatives more homogeneous and stable. At this regard, an NGR-TNF product lacking the +58 Da and +42 modifications can be obtained by expressing the cDNA encoding this protein in *P. pastoris*, instead of *E. coli* [62]. Furthermore, blockade of the cysteine α-amino group with a serine in first position leads to the expression in *E. coli* of a SCNGRCG-TNF product (called S-NGR-TNF) more homogeneous and stable than NGR-TNF [62]. Finally, a stable nano-formulations of NGR-TNF can be produced using TNF-bearing gold nanoparticles functionalized with a cyclic NGR-peptide having a N-methylated glycine in place of glycine, which completely prevents asparagine deamidation without impairing CD13 recognition [64]. All these products, tested only in animal models so far, may represent more homogeneous, stable, and active second-generation NGR-TNF that deserve further investigation. 

### 4.5. Other TNF-Based Tumor Targeting Agents

The results of preclinical and clinical studies obtained with NGR-TNF indicate that extremely low doses of this drug (e.g., 100 pg/mouse or 0.8 µg/m^2^ in patients) are sufficient to affect the tumor vasculature and to induce anti-tumor effects. This also suggests that the CNGRCG peptide (NGR) and the CD13 receptor represent an efficient ligand–receptor system for delivering TNF to the tumor vasculature. However, besides CNGRCG, other ligands and delivery systems can be exploited for increasing the therapeutic index of TNF [12]. For example, a conjugate consisting of TNF coupled to the tumor vasculature-homing peptide CRGRRST was shown to increase tumor vessel stability, vascular perfusion, and T-cell infiltration in a mouse model of pancreatic endocrine tumors, when administered biweekly at doses of 2 μg [65]. The selective advantage of this drug over TNF was lost with a dose of 0.2 μg and no beneficial effects at all were observed with a dose of 0.2 ng [65].

Antibodies can also be exploited as targeting ligands. For example, the single-chain Fv fragment of L19, an antibody against the alternatively spliced EDB domain of fibronectin, has been fused to TNF by genetic engineering technology and used for delivering TNF to various types of solid tumors in animal models and in patients [66,67]. Of note, this product (called L19-TNF) has shown anti-tumor activity in a murine glioblastoma model [68] and is currently investigated in phase I/II trials in glioma patients at relatively high doses (10–13 µg/kg), as a single agent or combined with lomustine (clinicaltrials.gov; NCT03779230 and NCT04573192). Early findings in glioblastoma patients showed that L19-TNF monotherapy could decrease blood perfusion within the tumor, which was associated with increased tumor necrosis and T-cell infiltration [67,69]. Anti-CD13 antibodies have also been used as TNF vehicles [70,71]. An interesting strategy developed to reduce the systemic toxicity of antibody-TNF conjugates is based on the use of conjugates bearing mutations in the TNF moiety that decrease its affinity for TNF receptors, thereby reducing off-target effects on normal cells [70,71]. These de-potentiated TNF versions, thanks to their specific accumulation on the target molecules, such as alternatively spliced EDB domain of fibronectin or CD13, can regain their activity on targeted cells, e.g., by local avidity-driven receptor binding. For example, administration of high doses (375 µg/kg) of a depotentiated version of L19-TNF (I97A) to mice bearing subcutaneous WEHI-164 fibrosarcomas showed a more potent anti-tumor activity, without apparent toxicity, than the wild-type product [71]. Similarly, daily injection of a CD13-specific VHH single-domain antibody fused to a mutated (Y86F) single-chain murine TNF (50 µg/mouse) could selectively activate the tumor neovasculature in a murine melanoma model without detectable toxicity [70,71]. Although these approaches might reduce the systemic toxicity of conjugates, the antibody ligands, the linkers, the mutations, and the markedly high doses necessary to induce anti-tumor effects might negatively impact the immunogenicity of this class of compounds, a point that should be carefully considered.

Other approaches used by different investigators for delivering TNF to tumors are based on the use of nanomaterials capable of exploiting the “passive” targeting mechanism consequent to the “enhanced permeability and retention effects (EPR)” of neoplastic tissues [72,73], such as gold nanoparticles [74,75,76,77,78,79,80], superparamagnetic iron oxide nanoparticles decorated exosome [81], magnetite (Fe_3_O_4_) nanoparticles [82], dendritic mesoporous silica nanoparticles [83], lactoferrin-bearing polypropylenimine dendriplexes [83], carbon dot festooned and surface-passivated graphene-reinforced chitosan nanoparticles [84], and poly-lactic acid microspheres [85]. Notably, recent studies have shown that the functionalization of TNF-bearing gold nanoparticles with NGR- or isoDGR-containing ligands can enable also the “active” targeted delivery of extremely low, but pharmacologically active, doses of nanodrug (e.g., equivalent to 5 pg of biologically active TNF/mouse) to the tumor vasculature in murine models [64,86]. Finally, genetically modified viruses, bacteria, and bacteriophages, engineered to express TNF alone or in combination with other cytokines, have also been developed [87,88,89,90,91,92,93]. For example, a recent study performed in a murine model of human glioblastoma have shown that an RGD4C-directed hybrid virus of adeno-associated virus and phage (AAVP) can deliver the TNF gene to the tumors, and induce damage of tumor-associated neovessels and cell death [94].

All these strategies are promising, but their clinical utility in safely breaching the BBB in cancer patients is yet to be demonstrated.

## 5. Conclusions

The results of studies performed with NGR-TNF in animal models of solid tumors and in PCNSL patients suggest that coupling TNF with the CNGRCG peptide (NGR) is a valuable strategy for delivering an amount of cytokine to tumor blood vasculature sufficient to alter the biological barriers that limit drug penetration in cancer lesions, including the BBTB. Remarkably, the dose of NGR-TNF necessary for BBTB alteration in patients (0.8 µg/m^2^) is well-tolerated. The results of these studies have also shown that the use of an extremely low dose of NGR-TNF can overcome, on the one hand, the problem of TNF systemic toxicity, and, on the other hand, systemic counter-regulatory mechanisms that limit the therapeutic efficacy of this cytokine. This is made possible by the excellent accessibility of the CD13 target (the luminal side of the endothelial lining of tumor vasculature, thus not beyond the BBTB) and the high avidity of the interactions among NGR-TNF, CD13 and TNF-receptors, co-expressed on the endothelium of the tumor vasculature. The NGR targeting domain, besides delivering TNF effector molecules to CD13-positive tumor vessels, can also induce co-signaling mechanisms that impair the activation of pro-survival pathways by the TNF moiety, a mechanism that may contribute to the biological effects observed with extremely low doses of the fusion protein.

The low dose of drug necessary for BBTB breaching, the target accessibility, the small size and the poor (or null) immunogenicity of the NGR peptide, may represent important advantages compared to other ligands and vehicles so far developed for delivering TNF (protein or gene) to tumors, e.g., those based on antibodies, nanoparticles, viral agents, or genetically engineered cells, particularly in the case of therapies that need repeated treatments. 

In conclusion, the results achieved so far in animal models and in PCNSL patients suggest that targeted delivery of low amounts of NGR-TNF (or its more homogeneous derivatives) to CD13-positive tumor neovasculature represents a valuable strategy for breaching the BBTB and for enhancing anticancer drug delivery to neoplastic cells in patients with PCNSL. Further studies are warranted to assess the potential use of NGR-TNF (or its more stable and homogeneous derivative S-NGR-TNF), in combination with chemo/immunotherapy, for the treatment of other primary and secondary brain tumors, such as glioblastoma multiforme and brain metastases of breast and lung cancer.
